# Determinants of clinical pregnancy outcomes in women with diminished ovarian reserve undergoing repeated assisted reproductive technology cycles: a retrospective cohort analysis of 3,895 oocyte retrieval cycles

**DOI:** 10.3389/fendo.2026.1846798

**Published:** 2026-07-31

**Authors:** Xueluo Zhang, Yanhua Chen, Zhiping Zhang, Xianping Wang, Xueqing Wu, Yuese Yuan, Yi Gao, Xingyu Bi

**Affiliations:** 1 Center of Reproductive Medicine, Children’s Hospital of Shanxi, Women Health Center of Shanxi, Taiyuan, China; 2 Shanxi Medical University, Taiyuan, China; 3 School of Public Health, Shanxi Medical University, Taiyuan, China

**Keywords:** art, diminished ovarian reserve, inflection point effect, influencing factors, pregnancy success rate

## Abstract

**Objective:**

This study aimed to evaluate factors associated with the clinical pregnancy rate in women with diminished ovarian reserve (DOR) undergoing repeated assisted reproductive technology (ART) cycles.

**Methods:**

A retrospective analysis was conducted on 3,895 oocyte retrieval (also referred to as oocyte pickup, [OPU]) cycles and 1,545 frozen embryo transfer cycles from 899 women with DOR. Multivariate logistic regression was used to identify independent factors associated with clinical pregnancy. Restricted cubic spline models were applied to assess potential non-linear relationships between key variables and pregnancy outcomes. Stratified analyses were performed according to age, first-cycle oocyte yield, number of OPU cycles, and cumulative oocyte yield.

**Results:**

The median age of the study participants was 35 years. Multivariate logistic regression analysis demonstrated that increasing age (OR = 0.89) and the total number of OPU cycles (OR = 0.92) were independently associated with lower clinical pregnancy rates. In contrast, first-cycle oocyte yield (OR = 1.11) and cumulative oocyte yield (OR = 1.04) were independently associated with higher clinical pregnancy rates. Restricted cubic spline analysis identified non-linear associations between key variables and clinical pregnancy outcomes. In the overall cohort, the clinical pregnancy rate declined with increasing age beyond approximately 32years, while no further increase was observed when cumulative oocyte yield exceeded approximately 15. Stratified analyses demonstrated that among women aged<33 and 33 to 37 years, the clinical pregnancy rate increased with cumulative oocyte yield up to approximately 9 oocytes, after which a plateau was observed. In women who obtain 0–1 oocytes in the first cycle, the probability of successful pregnancy gradually decreases with age after about 34 years old. And similar to women who obtained ≥4 oocytes in the first cycle, pregnancy success rates also decrease with the increase of OPU cycles after about 4 OPU cycles. In women who obtained 2–3 oocytes in the first cycle, pregnancy success rates began to decline after around the age of 32. In women undergoing 1–2 OPU cycles, clinical pregnancy rates increased with cumulative oocyte yield up to approximately 7 oocytes and declined thereafter. Among women with 3–4 OPU cycles, clinical pregnancy rates increased with cumulative oocyte yield up to approximately 14 oocytes and decreased beyond this inflection point. When cumulative oocyte yield ranged from 6 to 10, clinical pregnancy rates increased up to approximately 32 years of age and declined thereafter. For cumulative oocyte yield ≥ 11, clinical pregnancy rates remained relatively stable before approximately 32 years of age and decreased subsequently.

**Conclusion:**

Advancing age is an independent risk factor associated with reduced clinical pregnancy rates in women with DOR, with approximately 32 years representing a critical inflection point for reproductive potential. A inflection point effect was observed between cumulative oocyte yield and clinical pregnancy rate. Moderate increases in oocyte yield are associated with improved clinical outcomes; however, the benefit diminishes beyond a certain inflection point.

## 1 Introduction

Diminished ovarian reserve (DOR) is characterized by a reduction in oocyte quantity and/or quality, resulting in impaired ovarian function and reduced fertility potential. It is typically associated with decreased anti-Müllerian hormone (AMH) levels, reduced antral follicle count (AFC), and elevated basal follicle-stimulating hormone (FSH) levels. DOR can be categorized as physiological, related to advanced age, or pathological, occurring independently of age (1). The reported prevalence of DOR ranges from approximately 10% to 35% (2). Various clinical interventions have been proposed to improve ovarian function, including estrogen–progesterone therapy, antioxidant supplementation (e.g., dehydroepiandrosterone, coenzyme Q10, and vitamin E), and emerging approaches such as *in vitro* activation of ovarian tissue, stem cell–based therapies, and platelet-rich plasma treatment. However, the clinical efficacy of these interventions remains limited. For women with DOR seeking pregnancy, attempts at natural conception or treatment with assisted reproductive technology (ART) are commonly pursued; however, the clinical pregnancy rate remains below 40% (3).

In women with DOR undergoing ART, multiple factors contribute to reduced oocyte yield and compromised oocyte quality, leading to a limited number of transferable embryos. Consequently, many women undergo repeated ovarian stimulation cycles to obtain an adequate number of embryos for transfer, despite persistently low clinical pregnancy rates and an increased risk of miscarriage. These factors may prolong treatment duration, increase financial burden, and contribute to psychological stress. However, the extent to which repeated ovarian stimulation and embryo transfer cycles improve clinical pregnancy outcomes in this population remains unclear. This study retrospectively analyzed clinical data from multiple ovarian stimulation cycles to identify factors associated with pregnancy success rates in women with DOR undergoing repeated ART treatment.

## 2 Materials and methods

### 2.1 Study participants

A retrospective analysis was conducted on clinical data from women with DOR who underwent *in vitro* fertilization (IVF) or intracytoplasmic sperm injection (ICSI) treatment at the Reproductive Center of Shanxi Provincial Maternity and Child Health Hospital between January 2021 and December 2024. The observation period extended from the initiation of the first ART cycle to the occurrence of the first clinical pregnancy.

Participants were stratified according to age at the first oocyte retrieval (also referred to as oocyte pickup, [OPU]) (<33, 33–37, and ≥38 years), first-cycle oocyte yield (0–1, 2–3, ≥4 oocytes), cumulative oocyte yield (< 6, 6–10, and ≥ 11), and total number of OPU cycles (1–2, 3–4, and ≥5). The patient selection process is illustrated in **Supplementary Figure 1**.

Clinical pregnancy was defined as the primary outcome measure. Restricted cubic spline (RCS) analysis was applied to evaluate both linear and non-linear associations between the aforementioned variables and clinical pregnancy outcomes. Potential inflection points were identified based on the morphological characteristics of the fitted curves. To mitigate the risk of Type I errors and overfitting due to multiple subgroup comparisons, we adopted a conservative interpretation strategy. We prioritized the consistency of effect directions and clinical plausibility over isolated statistical significance. Furthermore, to ensure adequate statistical power, categorical variables such as age, oocyte yield, and OPU cycles were consolidated into broader groups. Effect sizes with 95% confidence intervals are reported to reflect the precision of the estimates.

All participants provided informed consent, and the study protocol was approved by the Ethics Committee of Children’s Hospital of Shanxi, Women Health Center of Shanxi (No. IRB-SB-2021-002).

### 2.2 Inclusion and exclusion criteria

Women were included in accordance with the *Expert Consensus on Clinical Diagnosis and Treatment of Diminished Ovarian Reserve* if at least two of the following three criteria were met (4): ① AMH < 1.1 ng/mL; ② bilateral AFC < 5–7 follicles; ③ basal FSH ≥ 10 U/L on two consecutive menstrual cycles (measured on days 2–4 of the menstrual cycle).

Women were excluded if any of the following conditions were present: premature ovulation or failure of ovarian stimulation; use of donor oocytes; history of pelvic inflammatory disease; polycystic ovary syndrome; intrauterine adhesions or severe uterine malformations; recurrent miscarriage; endometriosis; hydrosalpinx; severe male factor infertility; malignancy; prior chemotherapy or radiotherapy; severe cardiovascular disease; active sexually transmitted infections; platelet dysfunction, moderate-to-severe thrombocytopenia, or coagulation disorders; or chromosomal abnormalities in either partner.

### 2.3 Research methodology

#### 2.3.1 Ovarian stimulation protocols

Individualized ovarian stimulation protocols were selected based on patient characteristics, including age, body mass index (BMI), AMH levels, hormonal profiles, and transvaginal ultrasound findings. The protocols included ultra-long, long, short, gonadotropin-only (Gn-only), mild stimulation, antagonist, progestin-primed ovarian stimulation (PPOS), luteal phase stimulation, and natural cycles, all conducted in accordance with standard clinical procedures. Final oocyte maturation was triggered when follicular monitoring demonstrated either two leading follicles ≥ 18 mm or at least three follicles ≥ 17 mm. Triggering was achieved using one of the following regimens: (1) human chorionic gonadotropin (hCG; Livzon Pharmaceutical, 4,000–8,000 IU, subcutaneous injection); (2) recombinant hCG (Ovidrel, Merck Serono, 250 μg); or (3) triptorelin acetate (Decapeptyl, Ipsen, 0.2 mg). Transvaginal OPU was performed under ultrasound guidance 36 to 38 hours after triggering. Fertilization (IVF or ICSI) was selected based on semen parameters, followed by *in vitro* embryo culture for 72 hours. Decisions regarding embryo cryopreservation or extended culture to the blastocyst stage were based on embryo morphological grading and patient-specific clinical considerations.

#### 2.3.2 Endometrial preparation for frozen embryo transfer

Endometrial preparation was performed using natural, artificial, down-regulation, or ovarian stimulation cycles, depending on patient characteristics. When endometrial thickness reached ≥8 mm, as confirmed by transvaginal ultrasound, luteal transformation was initiated using oral dydrogesterone (20 mg twice daily) combined with either vaginal progesterone gel (Crinone, Merck Serono, 90 mg daily) or intramuscular progesterone (Zhejiang Xianju Pharmaceutical, 40 mg daily) for 3 to 5 days prior to embryo transfer.

#### 2.3.3 Embryo transfer and luteal phase support

Starting on the day of OPU, participants received luteal phase support with either oral allylestrenol (10 mg twice daily) for 3–5 days or intramuscular progesterone (40 mg once daily; 20 mg/vial) for 3 to 5 days.

Based on individual clinical conditions and institutional protocols, one or two cleavage-stage embryos or blastocysts were transferred. Luteal phase support was continued after embryo transfer until confirmation of non-pregnancy or until 10 weeks of gestation in cases of confirmed pregnancy, at which point treatment was discontinued. Clinical pregnancy was defined as the presence of an intrauterine gestational sac detected by transvaginal ultrasound approximately 28 days after embryo transfer.

#### 2.3.4 Statistical analysis

To avoid the potential non-independence of data arising from repeated oocyte retrieval (OPU) cycles within the same patient, the unit of statistical analysis in this study was defined at the patient level rather than the cycle level. Although a total of 3,895 OPU cycles were included (**Table 1**), the data were aggregated at the patient level during the preprocessing stage. Each patient contributed only a single, unique record to the final analytical dataset. Specifically, the independent variable, “number of oocyte retrieval cycles,” was defined as the cumulative number of cycles experienced by each patient, while the dependent variable was the final pregnancy outcome. Since each patient contributed only one observation to the model, the assumption of independence was satisfied. Therefore, standard binary logistic regression was employed to evaluate the association between the number of oocyte retrievals and pregnancy outcomes.

**Table 1:** The overall profiles of 899 female patients.

Characteristics	Total subjects (n = 899)
Infertility type (n, %)	
Primary infertility	415 (46.16%)
Secondary infertility	474 (53.84%)
Number of IVF/ICSI cycles (n, %)	
IVF	1233 (31.66%)
ICSI	1473 (37.82%)
others	1189 (30.53%)
Number of oocyte retrieval cycles	3895
Number of women having different oocyte retrieval cycles	
1	141 (15.68%)
2	182 (20.24%)
3	141 (15.68%)
4	129 (14.35%)
5	89 (9.90%)
≥6	217 (24.14%)
Number of FET cycles	1545
Number of women having different FET cycles	
0	149 (16.57%)
1	338 (37.60%)
2	213 (23.69%)
3	110 (12.24%)
4	46 (5.12%)
5	20 (2.22%)
≥6	23 (2.56%)
Median time duration of women having different OPU cycles (months)	
1	0.00 (0.00, 0.00)
2	3.00 (2.00, 5.25)
3	8.00 (4.00, 12.00)
4	10.00 (6.00, 14.50)
5	10.00 (7.50, 16.50)
≥6	22.00 (15.00, 38.50)
Median time duration of women having different FET cycles (months)	
1	5.00 (2.00, 10.00)
2	11.00 (6.00, 17.00)
3	16.00 (11.00, 23.00)
4	20.50 (13.75, 39.50)
5	26.00 (19.25, 63.25)
≥6	64.00 (37.00, 88.00)

Numbers are n (%) for categorical parameters. Median time duration of women having different oocyte pick-up (OPU) and FET cycles, the average duration between the date of the last OPU or embryo transfer and the date of the first oocyte retrieval. IVF, in vitro fertilization; ICSI, intracytoplasmic sperm injection; FET, frozen embryo transfer; OPU, oocyte pick-up.

All statistical analyses and data visualization were performed using R (version 4.5.1) and SPSS (version 26.0). The normality of continuous variables was assessed using the Shapiro–Wilk test. Normally distributed variables were presented as mean ± standard deviation (SD) and were compared using one-way analysis of variance (ANOVA). Non-normally distributed variables were presented as median (interquartile range, IQR) and were compared using the Kruskal–Wallis test. Categorical variables were expressed as frequencies and percentages and were compared using the chi-square test or Fisher’s exact test, as appropriate. *Post hoc* pairwise comparisons were conducted using the Bonferroni correction. A two-sided *p* value < 0.05 was considered statistically significant.

## 3 Results

### 3.1 Patient characteristics

This study included 899 women who underwent a total of 3,895 oocyte pickup (OPU) cycles and 1,545 frozen embryo transfer (FET) cycles (**Table 1**). Among them, 415 (46.2%) were diagnosed with primary infertility and 474 (52.7%) with secondary infertility (**Table 1**). The median age was 35 years (interquartile range [IQR]: 31–39), with a median interval between the first and last oocyte retrieval of 8 months (IQR: 2.5–17) and a median BMI of 23.39 kg/m² (IQR: 21.09–28.04) (**Table 2**).

**Table 2:** Comparison of characteristics between women with successful pregnancies and those with unsuccessful pregnancies.

Characteristics	All subjects	Pregnancy (-)	Pregnancy (+)	*P*
BMI (kg/m^2^)	23.39 (21.09, 28.04)	23.44 (21.23, 25.59)	23.14 (21.00, 25.97)	0.89
Age (years)	35 (31, 39)	36 (32, 41)	33 (30, 36)	<0.01
Number of women in different age groups				<0.01
<33	307 (34.15%)	148 (48.21%)	159 (51.79%)	
33-37	302 (33.59%)	162 (53.64%)	140 (46.36%)	
≥38	290 (32.26%)	232 (80.00%)	58 (20.00%)	
OPU cycles per woman (n)	3.00 (2.00, 5.00)	4.00 (2.00, 6.00)	3.00 (2.00, 5.00)	0.01
Number of women having different OPU cycles				<0.05
1-2	323 (35.93%)	179 (55.42%)	144 (44.58%)	
3-4	270 (30.03%)	163 (60.37%)	107 (39.63%)	
≥5	306 (34.04%)	200 (65.36%)	106 (34.64%)	
Number of initial oocyte retrieval per women (n)	3.00 (1.00, 5.00)	2.00 (1.00, 4.00)	3.00 (2.00, 6.00)	<0.01
Number of women in different initial oocyte retrieval groups				<0.01
0-1	286 (31.81%)	205 (71.68%)	81 (28.32%)	
2-3	287 (31.92%)	186 (64.81%)	101 (35.19%)	
≥4	326 (36.26%)	151 (46.32%)	175 (53.68%)	
Time duration of total OPU cycles (months)	8.00 (2.50, 17.00)	8.00 (3.00, 16.00)	7.00 (2.00, 18.00)	0.04
Total oocytes retrieved per women (n)	8.00 (5.00, 13.00)	7.00 (4.00, 12.00)	9.00 (6.00, 14.00)	<0.01
FSH	13.30 (11.28, 17.40)	13.80 (11.30, 18.20)	13.00 (11.20, 16.40)	<0.01
AFC	4.00 (2.00, 5.00)	3.00 (2.00, 5.00)	4.00 (2.00, 5.00)	0.43

Non-normally distributed data are presented as medians (first quartile, third quartile) and compared via nonparametric Kruskal–Wallis test. Categorical variables are expressed as numbers (%) and tested using chi-square test or Fisher’s test. *P* < 0.05 indicates that there is a statistically significant difference between the groups.

BMI, body mass index; OPU, oocyte pick-up; FSH, follicle stimulating hormone; AFC, antral follicle count.

The proportions of women who underwent 1–2, 3–4, and ≥5 OPU cycles were 35.93%, 30.03%, and 34.04%, respectively (**Table 2**). Based on age at first retrieval, patients were categorized into three groups (<33, 33–37, and ≥38 years), accounting for 34.15%, 33.59%, and 32.26% of the cohort, respectively (**Table 2**). According to the number of oocytes retrieved in the first cycle, patients were divided into three groups (0–1, 2–3, ≥4 oocytes), representing 31.81%, 31.92%, and 36.26% of the population, respectively (**Table 2**).

Compared with the non-pregnancy group, women who achieved pregnancy were significantly younger (P<0.05), had undergone fewer OPU procedures (P<0.05), had a higher number of oocytes retrieved in the first cycle (P<0.05), had a shorter interval between retrievals (P<0.05), had a greater total number of oocytes retrieved (P<0.05), and had lower follicle-stimulating hormone levels (P<0.05). The detailed characteristics are presented in **Supplementary Appendix Table 1**.

### 3.2 Factors associated with pregnancy outcomes

**Figure 1:**
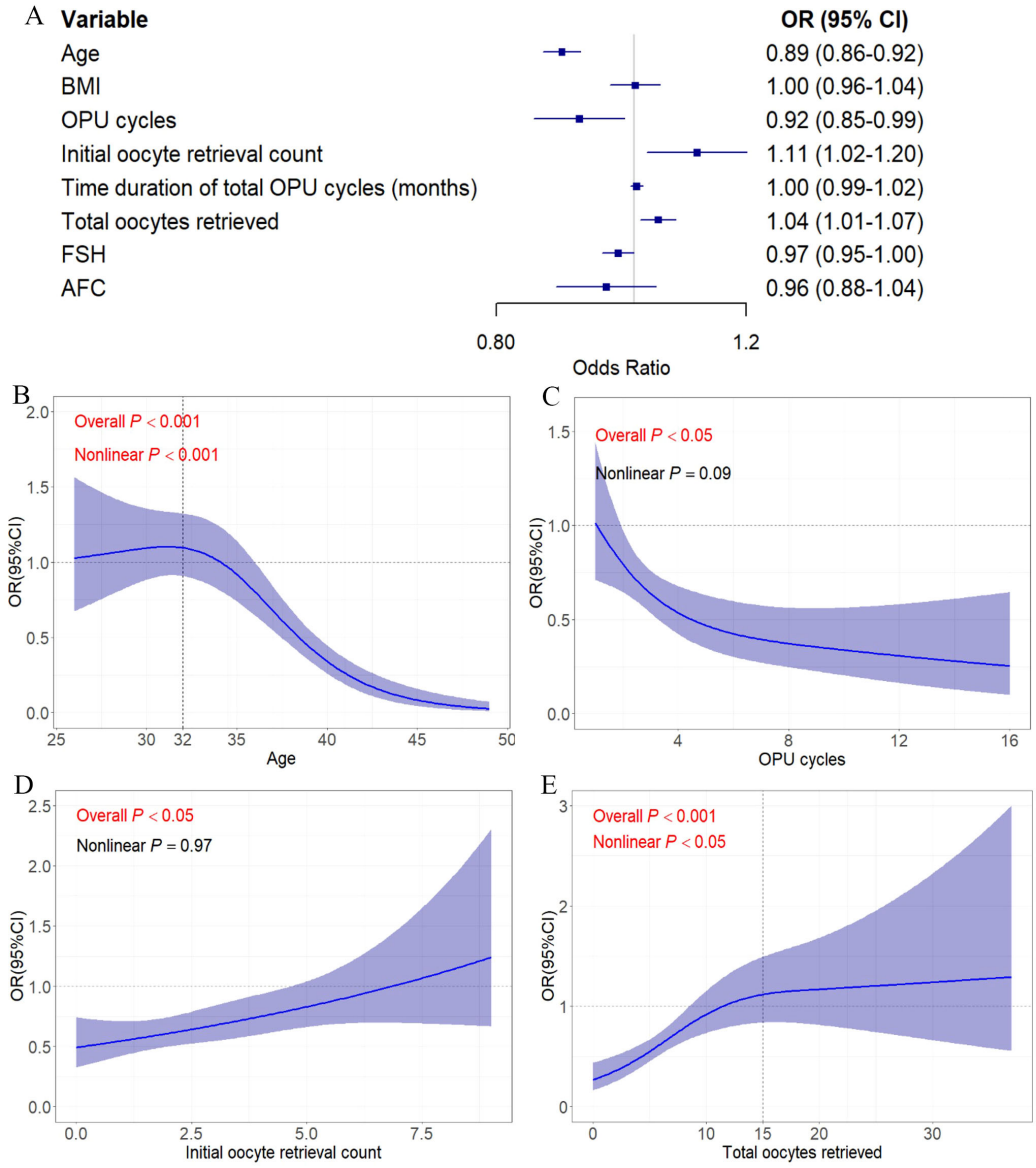
Association between multiple variables and pregnancy outcomes. **(A)** Forest plot displaying the overall associations. **(B–E)** Restricted cubic spline (RCS) curves exploring the potential linear and nonlinear relationships. Adjusted for age, BMI, OPU cycles, initial oocyte retrieval count, time duration of total OPU cycles, total oocytes retrieved, FSH and AFC. The solid line represents the adjusted OR, and the shaded area represents the 95% CI. BMI, body mass index; OPU, oocyte pick-up; FSH, follicle stimulating hormone; AFC, antral follicle count.

Multivariate logistic regression analysis, after adjusting for confounding factors, revealed that age and the number of OPU cycles were negatively correlated with the probability of successful pregnancy (P<0.05) (**Figure 1A**). In contrast, the number of oocytes retrieved in the first cycle and the total number of oocytes retrieved were positively correlated with pregnancy success (P<0.05) (**Figure 1A**).

Restricted cubic spline analyses indicated nonlinear relationships between both age and the total number of oocytes retrieved with the probability of pregnancy success (*P* for nonlinear *<* 0.05). Specifically, beyond approximately 32 years of age (OR: 1.10, 95%CI: 0.91-1.32), the probability of pregnancy success decreased with increasing age (**Figure 1B**). After a total oocyte count exceeded 15 (OR: 1.12, 95%CI: 0.84-1.49), further increases in oocyte number did not lead to a higher pregnancy success rate (**Figure 1E**).

### 3.3 Stratified analysis by age groups

Patients were stratified into three age groups based on their age at first oocyte retrieval: <33, 33–37, and ≥38 years. The ≥38 years group yielded the lowest number of oocytes retrieved in the first cycle and total number of oocytes (P<0.05) (**Supplementary Appendix Table 2**).

**Figure 2:**
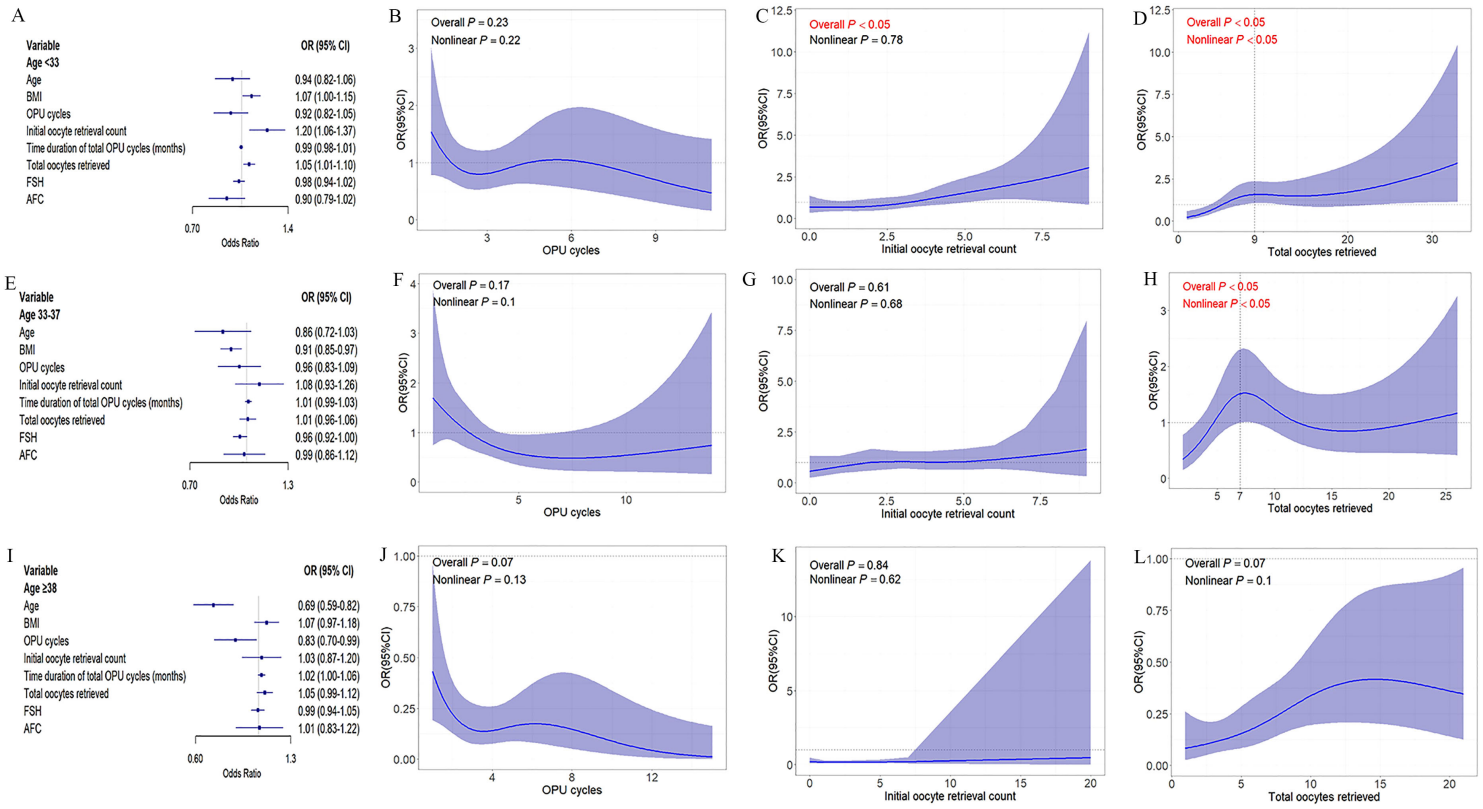
Association between multiple variables and pregnancy outcomes across pre-specified age subgroups. **(A, E, I)** Forest plot displaying the overall associations. **(B–D, F–H, J–L)** Restricted cubic spline (RCS) curves exploring the potential linear and nonlinear relationships. Adjusted for age, BMI, OPU cycles, initial oocyte retrieval count, time duration of total OPU cycles, total oocytes retrieved, FSH and AFC. The solid line represents the adjusted OR, and the shaded area represents the 95% CI. **(A–D)** represent the group with age <33. **(E–H)** represent the group with age 33-37. **(I–L)** represent the group with age ≥38. BMI, body mass index; OPU, oocyte pick-up; FSH, follicle stimulating hormone; AFC, antral follicle count.

Stratified analysis showed that for women aged <33 years, the number of oocytes retrieved in the first cycle and total number of oocytes were positively correlated with pregnancy success (P<0.05) (**Figure 2A**). A nonlinear relationship was observed between the total oocyte count and pregnancy success: when the total was less than around 9 (OR: 1.59, 95%CI: 1.07-2.37), the probability increased with oocyte number, but it plateaued thereafter (*P* for nonlinear *<* 0.05) (**Figure 2D**). For those aged 33–37 years, BMI was negatively correlated with success (P<0.05) (**Figure 2E**). The relationship between total oocyte yield and pregnancy success exhibited a nonlinear pattern. The likelihood of pregnancy rose as the oocyte count approached 9 (OR: 1.52, 95% CI: 0.99–2.31), but decreased beyond this inflection point. In the ≥38 years group, age and the number of OPU cycles were all negatively correlated with success (P<0.05) (**Figure 2I**).

### 3.4 Stratified analysis by the first-cycle oocyte yield

Stratification by first-cycle oocyte yield (0–1, 2–3, ≥4 oocytes; **Supplementary Appendix Table 3**) showed that the ≥4 oocytes group was the youngest and had the highest total oocyte count (P<0.05).

**Figure 3:**
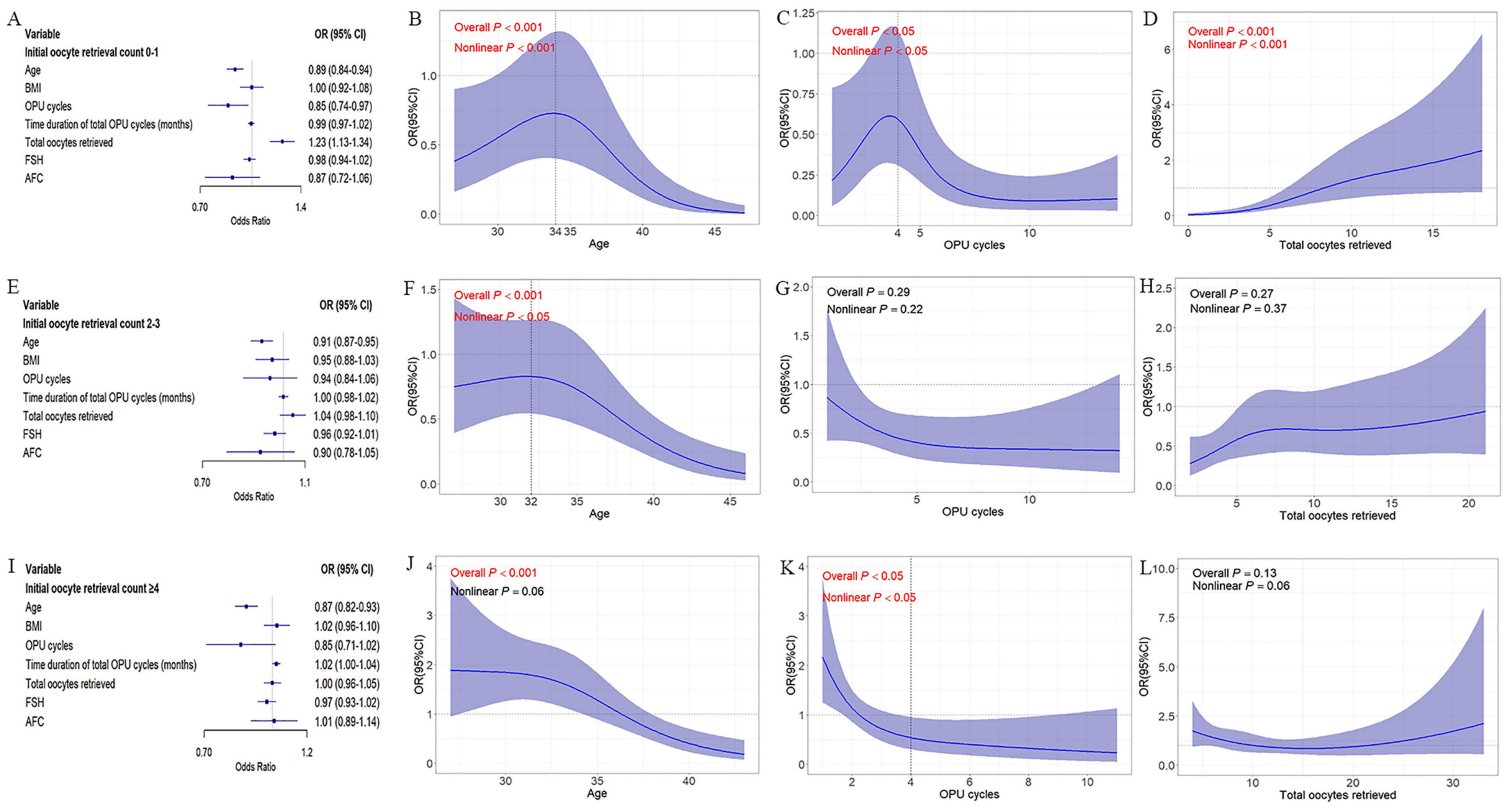
Association between multiple variables and pregnancy outcomes across subgroups stratified by the number of oocytes retrieved in the first cycle. **(A, E, I)** Forest plot displaying the overall associations. **(B–D, F–H, J–L)** Restricted cubic spline (RCS) curves exploring the potential linear and nonlinear relationships. Adjusted for age, BMI, OPU cycles, time duration of total OPU cycles, total oocytes retrieved, FSH and AFC. The solid line represents the adjusted OR, and the shaded area represents the 95% CI. **(A–D)** represent the group with initial oocytes retrieval count 0-1. **(E–H)** represent the group with initial oocytes retrieval count 2-3. **(I–L)** represent the group with initial oocytes retrieval count ≥4. BMI, body mass index; OPU, oocyte pick-up; FSH, follicle stimulating hormone; AFC, antral follicle count.

Age was negatively correlated with pregnancy success across all subgroups (P<0.05) (**Figures 3A, E, I**). In the 0–1 oocyte group, the number of OPU cycles was negatively correlated with success (P<0.05), while the total oocyte count was positively correlated (P<0.05). Age, OPU cycles, and total oocyte count exhibited nonlinear associations with the outcomes (**Figures 3B–D**). Approximately after the age of 34 (OR: 0.73, 95% CI: 0.40–1.32), as age increased, the probability of successful pregnancy decreased (*P* for nonlinear *<* 0.05, **Figure 3B**). The pregnancy success rate started to decrease beyond an inflection point of approximately 4 OPU cycles (OR: 0.59, 95% CI: 0.31–1.14) (*P* for nonlinear *<* 0.05, **Figure 3C**). In the 2–3 oocyte group, age showed nonlinear relationship with the outcome: the pregnancy success rate started to decrease beyond the inflection point of approximately 32 years (OR: 0.83, 95% CI: 0.54–1.26). (*P* for nonlinear *<* 0.05, **Figure 3F**). In the ≥4 oocyte group, the number of OPU cycles exhibited a nonlinear association with pregnancy outcomes. Specifically, the success rate decreased as the number of cycles increased up to 4 (OR: 0.54, 95% CI: 0.30–0.96), after which further increases in cycle numbers did not result in a significant additional decline. (*P* for nonlinear *<* 0.05, **Figure 3K**).

### 3.5 Stratified analysis by the number of OPU cycles

**Figure 4:**
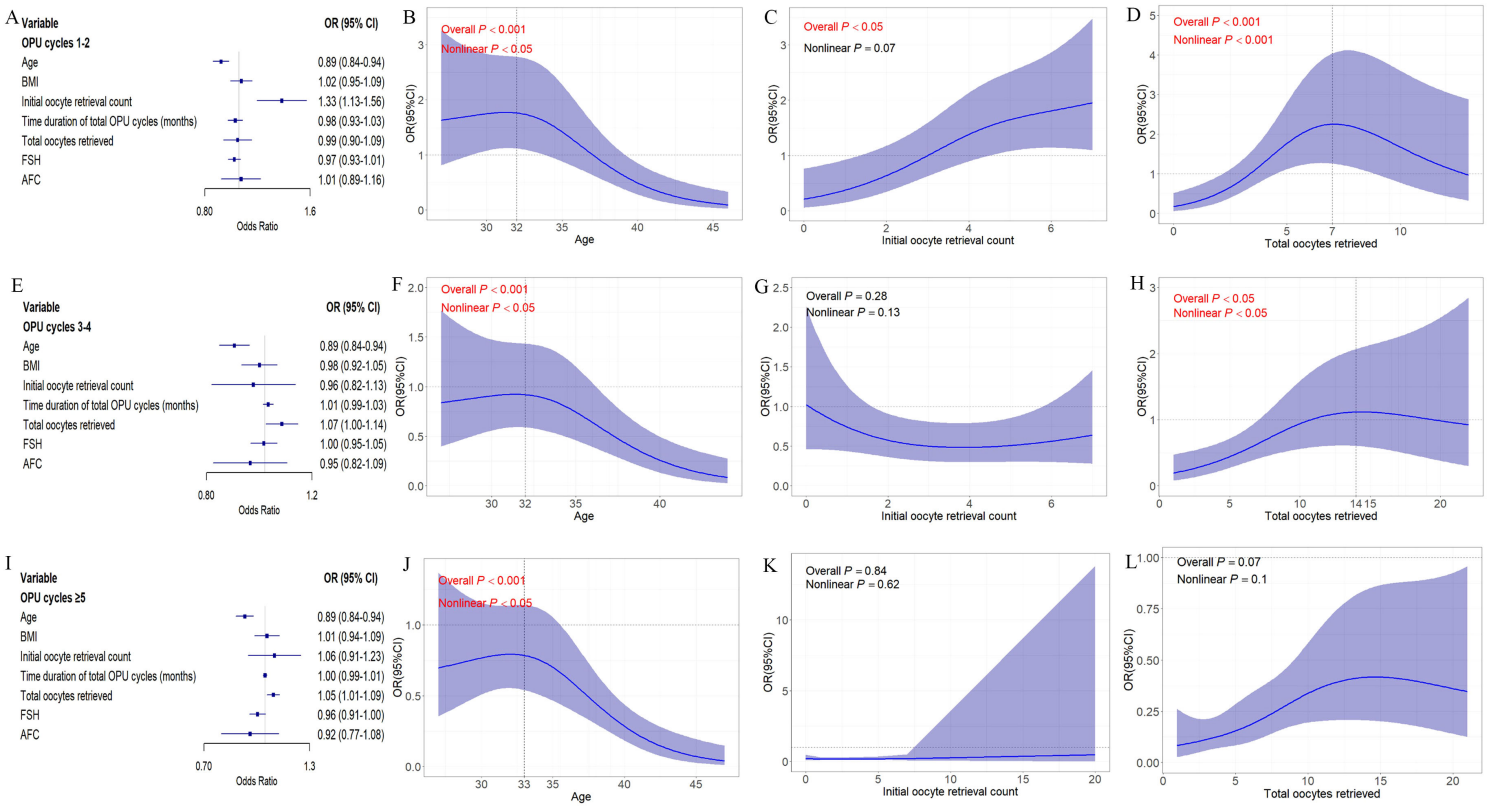
Association between multiple variables and pregnancy outcomes across subgroups stratified by OPU cycles. **(A, E, I)** Forest plot displaying the overall associations. **(B–D, F–H, J–L)** Restricted cubic spline (RCS) curves exploring the potential linear and nonlinear relationships. Adjusted for age, BMI, initial oocyte retrieval count, time duration of total OPU cycles, total oocytes retrieved, FSH and AFC. The solid line represents the adjusted OR, and the shaded area represents the 95% CI. **(A–D)** represent the group with OPU cycles 1-2. **(E–H)** represent the group with OPU cycles 3-4. **(I–L)** represent the group with OPU cycles ≥5. BMI, body mass index; OPU, oocyte pick-up; FSH, follicle stimulating hormone; AFC, antral follicle count.

When stratified by the number of OPU cycles (1–2, 3–4, ≥5; **Supplementary Appendix Table 4**), age was consistently negatively correlated with pregnancy success (P<0.05, **Figures 4A, E, I**).

In the group of OPU cycles 1–2, the first-cycle oocyte yield was positively correlated with success (P<0.05, **Figure 4A**). Age and the total oocyte count exhibited nonlinear associations with pregnancy outcomes (*P* for nonlinear *<* 0.05, **Figures 4B, D**). After an inflection point at 32 years of age (OR: 1.76, 95% CI: 1.12–2.78), pregnancy success rates started to decrease with advancing age, and the probability increased up to an inflection point of 7 oocytes (OR: 2.25, 95% CI: 1.26–4.03), beyond which further increases in oocyte yield were associated with a decline in success rates. In the group of OPU cycles 3–4, a nonlinear relationship was observed with age and the total oocyte count. After an inflection point at 32 years of age (OR: 0.92, 95% CI: 0.59–1.43), pregnancy success rates started to decrease with advancing age (**Figure 4F**). Success increased up to around 14 oocytes (OR: 1.12, 95% CI: 0.60–2.07), but decreased beyond that (*P* for nonlinear *<* 0.05) (**Figure 4H**). In the group of ≥5 OPU cycles, the total oocyte count was positively correlated with success. After an inflection point at 33 years of age (OR: 0.78, 95% CI: 0.54–1.14), pregnancy success rates started to decrease with advancing age (*P* for nonlinear *<* 0.05) (**Figure 4J**).

### 3.6 Stratified analysis by the total oocyte yield

**Figure 5:**
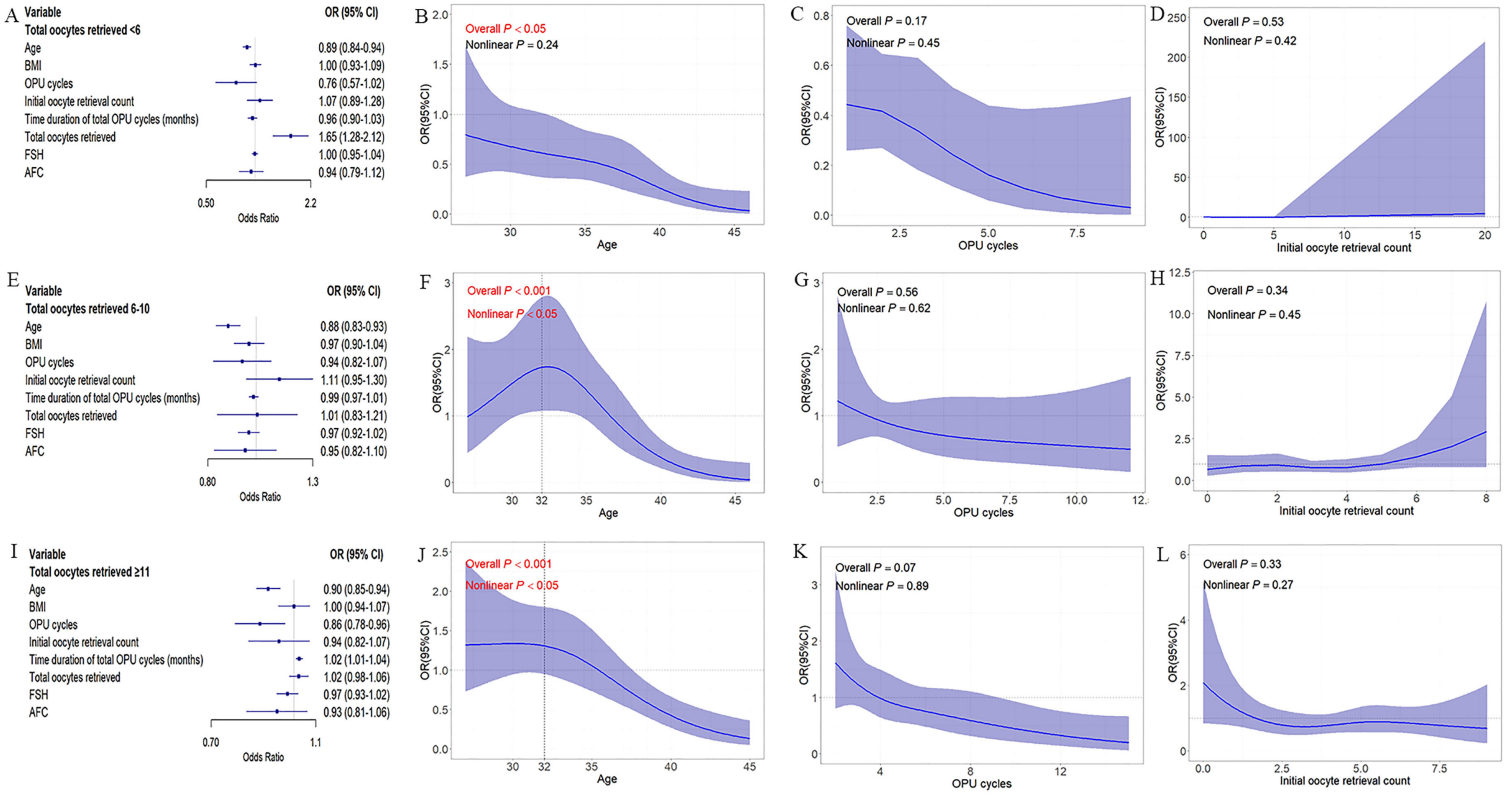
Association between multiple variables and pregnancy outcomes across subgroups stratified by the total number of oocytes retrieved. **(A, E, I)** Forest plot displaying the overall associations. **(B-D, F–H, J–L)** Restricted cubic spline (RCS) curves exploring the potential linear and nonlinear relationships. Adjusted for age, BMI, OPU cycles, initial oocyte retrieval count, time duration of total OPU cycles, total oocytes retrieved, FSH and AFC. The solid line represents the adjusted OR, and the shaded area represents the 95% CI. **(A–D)** represent the group with total oocytes retrieved <6. **(E–H)** represent the group with total oocytes retrieved 6-10.**(I–L)** represent the group with total oocytes retrieved ≥11. BMI, body mass index; OPU, oocyte pick-up; FSH, follicle stimulating hormone; AFC, antral follicle count.

Patients were categorized by the total oocyte count interquartile range into <6, 6–10, and ≥11 oocytes groups (**Supplementary Appendix Table 5**). Age was negatively correlated with success in all strata (P<0.05) (**Figures 5A, E, I**).

In the <6 oocytes group, the total oocyte count was positively correlated with success (P<0.05, **Figure 5A**), while in the ≥11 oocytes group, OPU cycles was negatively correlated (P<0.05, **Figure 5I**). In the 6–10 oocytes group, age had a nonlinear association with success: probability increased until around age 32 (OR: 1.73, 95% CI: 1.07–2.78), then declined (*P* for nonlinear *<* 0.05, **Figure 5F**). In the ≥11 oocytes group, success remained stable until approximately age 32 (OR: 1.31, 95% CI: 0.95–1.80), after which it began to decline with increasing age (*P* for nonlinear *<* 0.05, **Figure 5J**).

## 4 Discussion

Women with DOR often require multiple ovarian stimulation cycles to achieve satisfactory pregnancy outcomes due to reduced ovarian reserve and/or impaired oocyte quality. However, whether repeated IVF/ICSI-ET cycles confer consistent clinical benefit in this population remains uncertain, and the factors influencing treatment outcomes require further clarification. In this retrospective study of 3,895 OPU cycles in 899 women with DOR, factors associated with clinical pregnancy outcomes in the context of repeated ART cycles were systematically evaluated. The findings indicate that age, oocyte yield (including both first-cycle and cumulative yields), and the number of OPU cycles are key determinants of clinical pregnancy outcomes in this patient population.

### 4.1 Age is an independent risk factor for pregnancy outcomes in women with DOR

Age is a well-established independent determinant of outcomes in ART. Its impact on fertility is primarily mediated through declines in both oocyte quantity and quality, including increased rates of embryonic aneuploidy. Franasiak et al. analyzed 15,169 trophectoderm biopsy samples and reported an exponential increase in embryonic aneuploidy with advancing maternal age (5). Additionally, Morin et al. demonstrated that in women with DOR younger than 38 years, reduced ovarian reserve is predominantly characterized by a decline in oocyte quantity rather than quality (6).

In the present study, increasing age was consistently associated with lower clinical pregnancy rates in both overall and stratified analyses, in agreement with previous reports. Li et al. evaluated 900 women with DOR undergoing their first IVF/ICSI cycles and found that women who achieved pregnancy were significantly younger than those who did not (34.64 ± 0.43 vs. 38.16 ± 0.22 years, *p* < 0.001) (7). Multivariate analysis further indicated that women younger than 35 years had a 4.755-fold higher likelihood of achieving clinical pregnancy compared with those older than 40 years (adjusted OR = 4.755, 95% CI: 2.81–8.04, *p* < 0.001). Similarly, a multicenter retrospective study by Kobayashi et al. demonstrated that, among women with DOR, clinical pregnancy and live birth rates per embryo transfer cycle were more strongly associated with age than with AMH levels (8).

The present analysis further identified a non-linear relationship between age and clinical pregnancy outcomes. When cumulative oocyte yield ranged from 6 to 10, clinical pregnancy rates increased up to approximately 32 years of age and declined thereafter. When cumulative oocyte yield was ≥ 11, clinical pregnancy rates remained relatively stable until approximately 32 years of age and decreased with advancing age beyond this point. These findings indicate that approximately 32 years of age may represent a critical inflection point in reproductive potential for women with DOR.

For women younger than 32 years, accumulation of an adequate number of oocytes appears to be an important factor in improving clinical pregnancy outcomes. In contrast, for women of advanced age, particularly those aged ≥ 40 years, both reduced oocyte quantity and compromised oocyte quality contribute to limited reproductive potential. In such cases, the likelihood of achieving pregnancy remains constrained despite repeated OPU cycles, and alternative strategies, including individualized treatment approaches or consideration of oocyte donation, may be warranted based on clinical indications.

### 4.2 Complex non-linear relationship between the oocyte yield and pregnancy outcome

This study found that the number of oocytes obtained in the first cycle and the total number of retrieved oocytes were positively correlated with the success rate of pregnancy. This study identified a non-linear association between cumulative oocyte yield and clinical pregnancy outcomes, with distinct inflection point effects across subgroups. Among women aged<33 years, clinical pregnancy rates increased with cumulative oocyte yield when the total number of oocytes was < 9 and plateaued once this inflection point was reached. In women undergoing 1–2 OPU cycles, clinical pregnancy rates increased when cumulative oocyte yield was ≤ 7 and decreased when it exceeded this level. In women undergoing 3–4 OPU cycles, the success rate of pregnancy reaches its peak when the cumulative oocyte count is about 14, and decreases beyond this value. In women undergoing ≥ 5 OPU cycles, clinical pregnancy rates increased with cumulative oocyte. In women with a cumulative oocyte yield < 6, cumulative oocyte yield was positively associated with clinical pregnancy. Similarly, among women with a first-cycle oocyte yield of 0 to 1, cumulative oocyte yield demonstrated a positive association with clinical pregnancy. In contrast, in women with a first-cycle oocyte yield ≥ 6, clinical pregnancy rates decreased when cumulative oocyte yield exceeded approximately 10, further supporting a non-linear relationship between these variables.

These findings indicate that the relationship between cumulative oocyte yield and clinical pregnancy outcomes is not linear and is influenced by both treatment-related and patient-specific factors. The observed patterns may reflect the combined effects of a “denominator effect” and underlying patient heterogeneity in the context of cumulative ART treatment in women with DOR.

Moderate increases in cumulative oocyte yield may improve clinical pregnancy outcomes. Women with DOR typically have a limited number of oocytes retrieved per cycle; therefore, accumulation of oocytes across multiple OPU cycles may increase the likelihood of obtaining transferable and high-quality embryos. Previous studies have demonstrated that women with ≤ 3 oocytes retrieved have significantly lower clinical pregnancy rates in fresh cycles (9). Similarly, in frozen embryo transfer cycles, low oocyte yield has been associated with reduced clinical pregnancy rates (10). However, evidence also indicates that women who did not obtain transferable embryos in initial OPU cycles may still achieve a cumulative live birth rate of approximately 36% following continued IVF treatment (11). Conversely, other studies indicate that in women with no oocytes retrieved in the first OPU cycle, baseline clinical parameters may not reliably predict pregnancy outcomes in subsequent IVF cycles (12).

However, once cumulative oocyte yield exceeds a certain inflection point, further increases may not improve clinical pregnancy rates and may even be associated with reduced outcomes in some subgroups. One possible explanation is the “denominator effect.” A higher cumulative oocyte yield may reflect an increased total number of OPU cycles or prolonged treatment duration. During extended and repeated ovarian stimulation, advancing age may adversely affect oocyte quality and endometrial receptivity, thereby offsetting the potential benefits associated with increased oocyte yield.

In the present study, women aged ≥ 38 years underwent the highest total number of OPU cycles and experienced the longest treatment duration, yet had the lowest cumulative oocyte yield. This observation reflects a pattern in which prolonged treatment is associated with limited improvement in oocyte yield. In addition, patient-specific factors may act as confounders. Underlying fertility-limiting conditions, including embryonic, endometrial, or immunological factors, may contribute to variability in oocyte quality and reproductive outcomes in women with DOR. Although some studies indicate that oocyte quality in women with DOR may be comparable to that in women without DOR, heterogeneity is likely present depending on the underlying etiology. In some women, both oocyte quantity and quality may be compromised, resulting in an apparently adequate oocyte yield but suboptimal clinical pregnancy outcomes.

The inflection point relationship between cumulative oocyte yield and clinical pregnancy outcomes observed in this study has important clinical implications. For women with DOR aged<33 years, a cumulative yield of approximately 9 oocytes may represent a minimum target inflection point, beyond which additional OPU is associated with diminishing clinical benefit. For women undergoing 1–2 OPU cycles, a cumulative yield of approximately 7 oocytes may serve as a reference target. In women undergoing 3–4 OPU cycles, a cumulative yield of approximately 14 oocytes may be considered an indicative target. These findings may assist clinicians in optimizing treatment strategies by balancing potential clinical benefits with the time burden, financial cost, and psychological impact associated with repeated ART cycles.

### 4.3 Relationship between the number of OPU cycles and the pregnancy success rate

Previous studies have demonstrated that, in women aged ≥ 35 years—particularly those aged 35 to 43 years—increasing the number of IVF cycles to 3 to 4 is associated with a significant improvement in cumulative live birth rates (13, 14). In women with DOR aged < 40 years, each additional OPU cycle has been associated with an approximate 2% increase in cumulative live birth rate, with optimal outcomes observed after 6 to 7 OPU cycles (15). However, advancing age substantially modifies this relationship. Compared with women aged 38 to 39 years, those aged 40 to 42 years exhibit an approximately 35% reduction in cumulative live birth rates after four cycles (13). In women aged ≥ 40 years, cumulative live birth rates decline further, with estimates of approximately 16.5% after multiple cycles (15). Collectively, these findings indicate that advancing age—particularly ≥ 40 years—represents a critical inflection point beyond which the benefit of repeated IVF/ICSI cycles becomes limited. Repeated treatment cycles may not fully compensate for the age-related decline in reproductive potential in this population (13, 15, 16).

This study demonstrated a negative association between the number of OPU cycles and clinical pregnancy rates. Specifically, in both the group with a first-cycle oocyte yield of 0 to 1 and the group with a cumulative oocyte yield ≥ 11, the total number of OPU cycles was negatively associated with clinical pregnancy. In women with a first-cycle oocyte yield ≥ 4, a non-linear relationship was observed: when the number of OPU cycles exceeded approximately 4, clinical pregnancy rates declined with further increases in cycle number (*p* for non-linearity < 0.05).

At first consideration, these findings may appear inconsistent with clinical observations that cumulative pregnancy rates increase with additional OPU cycles. However, this discrepancy reflects differences in outcome measures. The present analysis evaluated clinical pregnancy rates per OPU cycle rather than cumulative pregnancy rates. As the number of OPU cycles increases, treatment is more likely to be continued in women with poorer ovarian response and less favorable prognostic characteristics. Consequently, the probability of success in each subsequent cycle may decline.

Consistent with these findings, Wang et al. reported that, in women with DOR, cumulative embryo transfer following repeated OPUs across multiple OPU cycles did not significantly improve cumulative pregnancy or live birth rates (17). This evidence supports the observation that per-cycle clinical pregnancy rates may decrease with increasing numbers of OPU cycles.

These findings indicate that the number of OPU cycles should be carefully considered in clinical practice. For younger women with relatively preserved ovarian reserve and a favorable response in the initial cycle, multiple OPU cycles may be pursued to facilitate embryo accumulation. In contrast, for women of advanced age with diminished ovarian reserve and suboptimal responses across multiple OPU cycles, the potential benefit of continued treatment should be carefully evaluated. This approach may help minimize the physical burden, psychological stress, and financial cost associated with repeated ART interventions.

### 4.4 Clinical significance and limitations

The findings of this study indicate that women with DOR undergoing ART may benefit from individualized, stratified management based on age and ovarian reserve status. For women younger than 32 years, accumulation of embryos through multiple OPU cycles may be associated with improved cumulative pregnancy outcomes, and this approach may be considered in appropriate clinical contexts. For women aged 33 to 37 years, the potential benefits of continued treatment should be balanced against time-related considerations, with careful evaluation of the number of treatment cycles. In women aged ≥ 38 years, the substantial impact of age on reproductive outcomes should be clearly communicated, and alternative options, including oocyte donation, may be considered when clinically appropriate.

Although this study demonstrated an association between oocyte yield and clinical pregnancy outcomes, emerging evidence indicates that oocyte quality in women with DOR may be comparable to that in women without DOR, with no significant differences in aneuploidy rates. In women from whom viable embryos are obtained, reproductive outcomes appear to be more strongly influenced by age than by ovarian reserve status alone. Accordingly, clinical management should consider both oocyte quantity and embryo quality, rather than focusing exclusively on increasing oocyte yield.

It is important to interpret these findings with caution. The inflection points identified (e.g., 32 years of age) are data-driven exploratory results. Given the confidence intervals and potential model sensitivity, these points should not be regarded as absolute clinical inflection points for decision-making. Instead, they serve as a reference to aid in individualized risk assessment and counseling.

As a retrospective study, selection bias is inevitable. Patients who underwent repeated OPU cycles might differ from those who discontinued treatment in terms of prognosis or motivation. We did not compare the baseline characteristics between patients who continued and those who stopped treatment; thus, our results regarding the limited benefit of additional cycles should be interpreted with this potential bias in mind. Several limitations regarding the subgroup analyses should be noted. First, the multiple comparisons performed in this study increase the probability of Type I errors. Although we merged categories to improve statistical power, some significant associations observed in smaller subgroups may be due to chance. Therefore, these results should be viewed as exploratory and hypothesis-generating. Future studies with larger, independent cohorts are needed to validate these findings, ideally with pre-specified subgroup hypotheses to minimize data-driven bias. In addition, the analysis focused on clinical pregnancy outcomes without evaluating live birth or cumulative live birth rates, which may be more clinically relevant endpoints for women with DOR.

## 5 Conclusions

This study, based on a large sample of 3,895 OPU cycles, systematically evaluated factors associated with clinical pregnancy outcomes in women with DOR undergoing repeated ART cycles, with particular emphasis on their non-linear relationships. Age was identified as an independent factor associated with clinical pregnancy outcomes, with approximately 32 years representing a potential inflection point in reproductive potential. A non-linear relationship was observed between cumulative oocyte yield and clinical pregnancy, with distinct inflection point effects across subgroups defined by age and number of OPU cycles.

These findings may provide a reference for individualized OPU targets in clinical practice. In addition, the number of OPU cycles was negatively associated with clinical pregnancy rates, which may reflect underlying selection bias and diminishing per-cycle benefit with repeated treatment. Overall, these findings may support the development of individualized treatment strategies and more precise prognostic counseling for women with DOR undergoing ART.

## Data Availability

The raw data supporting the conclusions of this article will be made available by the authors, without undue reservation.

## Supplementary material

Supplementary Figure 1 Participant enrollment and exclusion flow chart for ART cycles in women with DOR.
