# Supplementary material for: Determinants of clinical pregnancy outcomes in women with diminished ovarian reserve undergoing repeated assisted reproductive technology cycles: a retrospective cohort analysis of 3,895 oocyte retrieval cycles
Source: Front Endocrinol (Lausanne). 2026 Jul 31;17:1846798. doi: 10.3389/fendo.2026.1846798 (PMC13472783; doi:10.3389/fendo.2026.1846798)
Supplement: Supplementary file 1 [file DataSheet1.docx]

**Appendix Table1**

| Different outcome groups | Pregnancy (-) | | Pregnancy (+) | | | |
| --- | --- | --- | --- | --- | --- | --- |
|  | Embryo (-) | Embryo (+) | Live Birth (-) | | Live Birth (+) | |
|  |  |  | Embryo (-) | Embryo (+) | Embryo (-) | Embryo (+) |
| Age (years) | 37.00 (32.00, 42.00) ^a^ | 36.00 (32.00, 40.00) ^a^ | 34.00 (30.00, 36.00) ^b, c^ | 35.00 (30.00, 39.00) ^c^ | 31.00 (29.00, 35.00) ^b^ | 33.00 (31.00, 35.00) ^b, c^ |
| BMI (kg/m^2^) | 23.69 (20.94, 25.58) | 23.12 (21.48, 25.63) | 23.70 (21.27, 27.62) | 22.86 (21.22, 25.78) | 23.01 (20.51, 25.89) | 23.19 (20.98, 25.80) |
| Number of women having different OPU cycles ^*^ | |  |  |  |  |  |
| 1 | 53 (21.03%) | 20 (6.90%) | 3 (8.57%) | 4 (6.90%) | 25 (29.76%) | 36 (20.00%) |
| 2 | 56 (22.22%) | 50 (17.24%) | 7 (20.00%) | 8 (13.79%) | 21 (25.00%) | 40 (22.22%) |
| 3 | 36 (14.29%) | 49 (16.90%) | 6 (17.14%) | 7 (12.07%) | 12 (14.29%) | 31 (17.22%) |
| 4 | 32 (12.70%) | 46 (15.86%) | 5 (14.29%) | 8 (13.79%) | 8 (9.52%) | 30 (16.67%) |
| 5 | 25 (9.92%) | 34 (11.72%) | 5(14.29%) | 6 (10.34%) | 6 (7.14%) | 13 (7.22%) |
| ≥6 | 50 (19.84%) | 91 (31.38%) | 9 (25.71%) | 25 (43.10%) | 12 (14.29%) | 30 (16.67%) |
| Time duration of total OPU (months） | 6.00 (1.00, 13.00) ^a^ | 10.00 (4.00, 17.00) ^b^ | 15.00 (6.00, 52.00) ^c^ | 17.00 (7.00, 33.00) ^c^ | 3.00 (0.00, 13.50) ^a^ | 6.00 (1.50, 13.00) ^a^ |
| Number of women on different ovulation induction protocols, n (%) ^*^ | | |  |  |  |  |
| GnRH antagonist | 319 (32.95%) | 466 (30.80%) | 49 (27.07%) | 123 (37.61%) | 82 (29.71%) | 212 (33.65%) |
| Mild stimulation | 131 (13.53%) | 180 (11.90%) | 24 (13.26%) | 25 (7.65%) | 27 (9.78%) | 67 (10.63%) |
| Ultralong protocol | 33 (3.41%) | 70 (4.63%) | 15 (8.29%) | 23 (7.03%) | 15 (5.43%) | 32 (5.08%) |
| Long protocol | 52 (5.37%) | 77 (5.09%) | 16 (8.84%) | 15 (4.59%) | 32 (11.59%) | 55 (8.73%) |
| Short protocol | 84 (8.68%) | 160 (10.58%) | 19 (10.50%) | 27 (8.26%) | 32 (11.59%) | 76 (12.06%) |
| GnRH agonist | 112 (11.57%) | 177 (11.70%) | 36 (19.89%) | 45 (13.76%) | 26 (9.42%) | 65 (10.32%) |
| PPOS related | 28 (2.89%) | 48 (3.17%) | 6 (3.31%) | 13 (3.98%) | 8 (2.90%) | 28 (4.44%) |
| Others | 209 (21.59%) | 335 (22.14%) | 16 (8.84%) | 56 (17.13%) | 54 (19.57%) | 95 (15.08%) |
| IVF/ICSI, n (%) ^*^ |  |  |  |  |  |  |
| IVF | 306 (31.61%) | 381 (25.18%) | 61 (33.70%) | 107 (32.72%) | 111 (40.22%) | 267 (42.38%) |
| ICSI | 260 (26.86%) | 622 (41.11%) | 76 (41.99%) | 167 (51.07%) | 83 (30.07%) | 265 (42.06%) |
| others | 402 (41.53%) | 510 (33.71%) | 44 (24.31%) | 53 (16.21%) | 82 (29.71%) | 98 (15.56%) |
| Number of initial oocyte retrieval per women (n) | 3.00 (1.00, 4.50) ^a^ | 3.00 (1.00, 5.00) ^b^ | 2.00 (1.00, 3.50) ^b, c^ | 2.50 (1.00, 4.00) ^b, c^ | 3.00 (1.00, 5.00) ^c^ | 2.00 (1.00, 4.00) ^c^ |
| Total oocytes retrieved per women (n) | 5.00 (2.00, 9.00) ^a^ | 9.00 (6.00, 14.00) ^b, c^ | 11.00 (5.50, 17.00) ^c, d^ | 13.00 (9.00, 18.00) ^d^ | 6.00 (4.00, 11.00) ^e^ | 9.00 (6.00, 13.50) ^c^ |
| Total embryos retrieved per women (n) | 2.00 (1.00, 3.00) ^a^ | 4.00 (3.00, 6.00) ^b, c^ | 4.00 (3.00, 6.50) ^c, d, f^ | 5.00 (4.00, 8.00) ^d^ | 2.00 (2.00, 4.00) ^e^ | 4.00 (3.00, 6.00) ^f^ |
| Cleavage embryos (n) | 2.00 (0.00, 3.00) ^a,^ ^d^ | 2.00 (0.00, 4.00) ^b, d, e^ | 3.00 (1.50, 5.00) ^c^ | 3.00 (1.00, 6.00) ^c^ | 2.00 (0.00, 3.00) ^d, e^ | 2.00 (0.00, 4.00) ^e^ |
| Blast embryos (n) | 0.00 (0.00, 0.00) ^a^ | 1.00 (0.00, 3.00) b, c | 1.00 (0.00, 2.50) c, d | 2.00 (0.00, 5.00) d, e | 1.00 (0.00, 2.00) c | 2.00 (1.00, 4.00) e |
| FET cycles per women (n) | 1.00 (1.00, 2.00) ^a^ | 1.00 (0.00, 2.00) a | 3.00 (2.00, 5.00) b | 2.00 (1.00, 3.00) b | 2.00 (1.00, 3.00) c | 1.00 (1.00, 2.00) c |
| FET protocol proportion, n（%） | |  |  |  |  |  |
| Artificial cycle | 146 (40.90%) | 180 (44.67%) | 58 (47.15%) | 63 (39.62%) | 67 (38.29%) | 158 (48.32%) |
| Natural cycle | 76 (21.29%) | 65 (16.13%) | 28 (22.76%) | 25 (15.72%) | 40 (22.86%) | 53 (16.21%) |
| Ovulation induction cycle | 73 (20.45%) | 81 (20.10%) | 20 (16.26%) | 39 (24.53%) | 41 (23.43%) | 59 (18.04%) |
| Down-regulation cycle | 62 (17.37%) | 77 (19.11%) | 17 (13.82%) | 32 (20.13%) | 27 (15.43%) | 57 (17.43%) |
| Average number of OPU cycles per embryo transfer | |  | 1.47 | 2.06 | 1.58 | 1.93 |
| First OPU- last transfer interval |  |  | 15.00 (9.50, 36.50) ^a^ | 18.00 (5.00, 25.00) ^a^ | 6.00 (3.00, 14.50) ^b^ | 9.00 (3.50, 14.00) ^b^ |
| First OPU-first clinical pregnancy interval | |  | 7.50 (3.50, 15.00) | 7.00 (3.50, 16.50) | 5.00 (3.00, 10.50) | 7.00 (3.00, 12.50) |
| Number of embryo transfers to first clinical pregnancy | |  | 1.00 (1.00, 3.00) | 1.00 (1.00, 2.00) | 1.00 (1.00, 2.00) | 1.00 (1.00, 2.00) |
| Number of embryos to first clinical pregnancy | |  | 2.00 (2.00, 4.00) | 2.00 (1.00, 4.00) | 2.00 (2.00, 3.00) | 2.00 (1.00, 3.00) |
| First OPU- first live birth interval | |  |  |  | 6.00 (3.00, 12.00) | 8.50 (3.00, 13.00) |
| Number of embryo transfers to live birth | |  |  |  | 1.00 (1.00, 2.00) | 1.00 (1.00, 2.00) |
| Number of embryos to live birth | |  |  |  | 2.00 (2.00, 4.00) | 2.00 (1.00, 3.00) |

The characteristics of women in different outcome groups. Non-normally distributed continuous data are presented as median (interquartile range, IQR) and compared using the Kruskal-Wallis test. Categorical variables are expressed as number (%) and compared using the chi-square or Fisher’s exact test. For post-hoc pairwise comparisons, the Bonferroni correction was applied. Different superscript letters (a, b, c, d, e and f) and asterisk (*) indicate statistically significant differences between groups at *P*< 0.05. BMI, body mass index; OPU, oocyte pick-up; GnRH, gonadotropin-releasing hormone; PPOS, progestin-primed ovarian stimulation; IVF, *in vitro* fertilization; ICSI, intracytoplasmic sperm injection; ‌FET, frozen embryo transfer.

**Appendix Table2**

| Age groups | Group1 <33 (n=307) | Group2 33-37 (n=302) | Group3 ≥ 38 (n=290) |
| --- | --- | --- | --- |
| BMI (kg/m^2^) | 23.83 (21.23, 26.45) | 22.66 (20.42, 24.71) | 23.01 (21.99, 25.32) |
| Number of women having different OPU cycles | |  |  |
| 1 | 52 (16.94%) | 40 (13.25%) | 49 (16.90%) |
| 2 | 67 (21.82%) | 67 (22.19%) | 48 (16.55%) |
| 3 | 50 (16.29%) | 58 (19.21%) | 33 (11.38%) |
| 4 | 43 (14.01%) | 41 (13.58%) | 45 (15.52%) |
| 5 | 21 (6.84%) | 30 (9.93%) | 38 (13.10%) |
| ≥6 | 74 (24.10%) | 66 (21.85%) | 77 (26.55%) |
| Time duration of total OPU (months） | 4.00 (0.00, 16.00) | 5.00 (2.00, 11.50) | 5.00 (0.00, 10.75) |
| Number of women on different ovulation induction protocols, n (%) ^*^ | | |  |
| GnRH antagonist | 369 (30.02%) | 380 (29.85%) | 502 (36.04%) |
| Mild stimulation | 138 (11.23%) | 162 (12.73%) | 154 (11.06%) |
| Ultralong protocol | 74 (6.02%) | 72 (5.66%) | 42 (3.02%) |
| Long protocol | 104 (8.46%) | 103 (8.09%) | 40 (2.87%) |
| Short protocol | 128 (10.41%) | 136 (10.68%) | 134 (9.62%) |
| GnRH agonist | 159 (12.94%) | 160 (12.57%) | 142 (10.19%) |
| PPOS related | 37 (3.01%) | 46 (3.61%) | 48 (3.45%) |
| Others | 220 (17.9%) | 214 (16.81%) | 331 (23.76%) |
| IVF/ICSI, n (%) ^*^ |  |  |  |
| IVF | 435 (35.39%) | 420 (32.99%) | 378 (27.14%) |
| ICSI | 472 (38.41%) | 513 (40.3%) | 488 (35.03%) |
| others | 322 (26.2%) | 340 (26.71%) | 527 (37.83%) |
| Number of initial oocyte retrieval per women (n) | 3.00 (1.00, 5.00) ^a^ | 3.00 (1.00, 5.00) ^a^ | 2.00 (1.00, 3.00) ^b^ |
| Total oocytes retrieved per women (n) | 8.00 (5.00, 15.00) ^a^ | 8.00 (6.00, 13.00) ^a^ | 7.50 (4.00, 11.50) ^b^ |
| Total embryos retrieved per women (n) | 4.00 (3.00, 6.00) ^a^ | 4.00 (3.00, 6.00) ^a^ | 3.00 (2.00, 5.00) ^b^ |
| Cleavage embryos (n) | 2.00 (0.00, 3.00) | 2.00 (0.00, 4.00) | 1.00 (0.00, 2.75) |
| Blast embryos (n) | 2.00 (0.00, 3.00) ^a^ | 2.00 (0.00, 3.00) ^a^ | 1.50 (0.25, 3.00) ^b^ |
| FET cycles per women (n) | 2.00 (1.00, 3.00) ^a^ | 1.00 (1.00, 2.00) ^a^ | 1.00 (1.00, 2.00) ^b^ |
| FET protocol proportion, n（%）^*^ |  |  |  |
| Artificial cycle | 248 (41.26%) | 235 (45.72%) | 189 (44.06%) |
| Natural cycle | 124 (20.63%) | 75 (14.59%) | 88 (20.51%) |
| Ovulation induction cycle | 133 (22.13%) | 102 (19.84%) | 78 (18.18%) |
| Down-regulation cycle | 96 (15.97%) | 102 (19.84%) | 74 (17.25%) |
| Average number of OPU cycles per embryo transfer | 2.05 | 2.48 | 3.24 |
| First OPU- last transfer interval | 9.00 (3.00, 17.50) | 9.00 (4.00, 13.00) | 7.50 (3.00, 13.50) |
| First OPU-first clinical pregnancy interval | 6.00 (3.00, 12.00) | 7.00 (3.00, 11.00) | 7.00 (3.00, 12.00) |
| Number of embryo transfers to first clinical pregnancy | 2.00 (1.00, 3.00) | 1.00 (1.00, 2.00) | 1.00 (1.00, 2.00) |
| Number of embryos to first clinical pregnancy | 0.00 (0.00, 2.00) ^a^ | 0.00 (0.00, 2.00) ^a^ | 0.00 (0.00, 0.00) ^b^ |
| First OPU- first live birth interval | 7.00 (3.00, 13.00) | 8.00 (4.00, 12.00) | 7.00 (3.00, 12.00) |
| Number of embryo transfers to live birth | 1.00 (1.00, 2.00) | 1.00 (1.00, 2.00) | 1.00 (1.00, 2.00) |
| Number of embryos to live birth | 0.00 (0.00, 2.00) ^a^ | 0.00 (0.00, 2.00) ^a^ | 0.00 (0.00, 0.00) ^b^ |

The characteristics of women in different age groups. Non-normally distributed continuous data are presented as median (interquartile range, IQR) and compared using the Kruskal-Wallis test. Categorical variables are expressed as number (%) and compared using the chi-square or Fisher’s exact test. For post-hoc pairwise comparisons, the Bonferroni correction was applied. Different superscript letters (a and b) and asterisk (*) indicate statistically significant differences between groups at *P* < 0.05. BMI, body mass index; OPU, oocyte pick-up; GnRH, gonadotropin-releasing hormone; PPOS, progestin-primed ovarian stimulation; IVF, *in vitro* fertilization; ICSI, intracytoplasmic sperm injection; ‌FET, frozen embryo transfer.

**Appendix Table3**

| Initial oocyte retrieval number groups | Group1 0-1 (n=286) | Group2 2-3 (n=287) | Group3 ≥4 (n=326) |
| --- | --- | --- | --- |
| Age (years) | 34.00 (31.00, 36.00) ^a^ | 34.00 (30.00, 36.00) ^a^ | 32.00 (30.00, 35.00) ^b^ |
| BMI (kg/m^2^) | 23.00 (21.48, 25.56) | 23.67 (20.28, 25.71) | 23.14 (21.00, 26.04) |
| Number of women having different OPU cycles ^*^ | |  |  |
| 1 | 24 (8.39%) | 29 (10.10%) | 88 (26.99%) |
| 2 | 38 (13.29%) | 61 (21.25%) | 83 (25.46%) |
| 3 | 37 (12.94%) | 42 (14.63%) | 62 (19.02%) |
| 4 | 44 (15.38%) | 52 (18.12%) | 33 (10.12%) |
| 5 | 42 (14.69%) | 26 (9.06%) | 21 (6.44%) |
| ≥6 | 101 (35.31%) | 77 (26.83%) | 39 (11.96%) |
| Time duration of total OPU (months） | 7.00 (4.00, 17.00) ^a^ | 5.00 (2.00, 12.00) ^a^ | 3.00 (0.00, 13.00) ^b^ |
| Number of women on different ovulation induction protocols, n (%) ^*^ | |  |  |
| GnRH antagonist | 517 (33.57%) | 409 (31.13%) | 325 (31.22%) |
| Mild stimulation | 221 (14.35%) | 169 (12.86%) | 64 (6.15%) |
| Ultralong protocol | 47 (3.05%) | 60 (4.57%) | 81 (7.78%) |
| Long protocol | 47 (3.05%) | 69 (5.25%) | 131 (12.58%) |
| Short protocol | 115 (7.47%) | 125 (9.51%) | 158 (15.18%) |
| GnRH agonist | 161 (10.45%) | 168 (12.79%) | 132 (12.68%) |
| PPOS related | 37 (2.4%) | 57 (4.34%) | 37 (3.55%) |
| Others | 395 (25.65%) | 257 (19.56%) | 113 (10.85%) |
| IVF/ICSI, n (%) ^*^ |  |  |  |
| IVF | 423 (27.47%) | 450 (34.25%) | 360 (34.58%) |
| ICSI | 436 (28.31%) | 489 (37.21%) | 548 (52.64%) |
| Others | 681 (44.22%) | 375 (28.54%) | 133 (12.78%) |
| Total oocytes retrieved per women (n) | 8.00 (5.00, 13.00) ^a^ | 7.00 (5.00, 11.00) ^b^ | 9.00 (6.00, 15.00) ^c^ |
| Total embryos retrieved per women (n) | 4.00 (3.00, 6.00) ^a^ | 3.50 (2.00, 5.00) ^b^ | 4.00 (3.00, 6.00) ^c^ |
| Cleavage embryos (n) | 2.00 (1.00, 4.00) | 2.00 (1.00, 3.00) | 1.00 (0.00, 3.00) |
| Blast embryos (n) | 1.00 (0.00, 3.00) ^a^ | 1.50 (0.00, 3.00) ^b^ | 2.00 (1.00, 4.00) ^c^ |
| FET cycles per women (n) | 1.00 (1.00, 3.00) ^a^ | 1.00 (1.00, 2.00) ^b^ | 2.00 (1.00, 2.00) ^c^ |
| FET protocol proportion, n (%) ^*^ |  |  |  |
| Artificial cycle | 157 (40.67%) | 198 (41.68%) | 317 (46.41%) |
| Natural cycle | 76 (19.69%) | 89 (18.74%) | 122 (17.86%) |
| Ovulation induction cycle | 91 (23.58%) | 90 (18.95%) | 132 (19.33%) |
| Down-regulation cycle | 62 (16.06%) | 98 (20.63%) | 112 (16.4%) |
| Average number of OPU cycles per embryo transfer | 3.99 | 2.76 | 1.52 |
| First OPU- last transfer interval | 11.00 (6.00, 15.00) ^a^ | 8.00 (4.00, 12.00) ^b^ | 6.00 (3.00, 14.00) ^b^ |
| First OPU-first clinical pregnancy interval | 10.00 (5.00, 14.00) ^a^ | 7.50 (4.00, 12.00) ^a^ | 5.00 (2.00, 10.00) ^b^ |
| Number of embryo transfers to first clinical pregnancy | 1.00 (1.00, 2.00) | 1.00 (1.00, 2.00) | 1.00 (1.00, 2.00) |
| Number of embryos to first clinical pregnancy | 0.00 (0.00, 1.00) ^a^ | 0.00 (0.00, 1.50) ^a^ | 1.00 (0.00, 2.00) ^b^ |
| First OPU- first live birth interval | 11.00 (6.00, 15.00) ^a^ | 8.00 (4.00, 12.00) ^a^ | 5.00 (2.00, 12.00) ^b^ |
| Number of embryo transfers to live birth | 1.00 (1.00, 2.00) | 1.00 (1.00, 2.00) | 1.00 (1.00, 2.00) |
| Number of embryos to live birth | 0.00 (0.00, 0.00) ^a^ | 0.00 (0.00, 0.00) ^a^ | 0.00 (0.00, 2.00) ^b^ |

The characteristics of women in different initial oocyte retrieval number groups. Non-normally distributed continuous data are presented as median (interquartile range, IQR) and compared using the Kruskal-Wallis test. Categorical variables are expressed as number (%) and compared using the chi-square or Fisher’s exact test. For post-hoc pairwise comparisons, the Bonferroni correction was applied. Different superscript letters (a, b and c) and asterisk (*) indicate statistically significant differences between groups at *P* < 0.05. BMI, body mass index; OPU, oocyte pick-up; GnRH, gonadotropin-releasing hormone; PPOS, progestin-primed ovarian stimulation; IVF, *in vitro* fertilization; ICSI, intracytoplasmic sperm injection; ‌FET, frozen embryo transfer.

**Appendix Table4**

| OPU cycle groups | Group1 1-2 (n=323) | Group2 3-4 (n=270) | Group3 ≥5 (n=306) |
| --- | --- | --- | --- |
| Age (years) | 33.00 (30.00, 36.00) | 33.00 (30.00, 35.00) | 33.00 (30.00, 35.00) |
| BMI (kg/m^2^) | 23.37 (21.00, 26.04) | 22.90 (20.70, 25.10) | 23.00 (20.70, 26.20) |
| Time duration of total OPU (months） | 1.00 (0.00, 3.00) ^a^ | 9.00 (6.00, 15.00) ^b^ | 17.00 (9.00, 29.00) ^c^ |
| Number of women on different ovulation induction protocols, n (%) ^*^ | | |  |
| GnRH antagonist | 193 (38.22%) | 323 (34.4%) | 735 (29.99%) |
| Mild stimulation | 36 (7.13%) | 91 (9.69%) | 327 (13.34%) |
| Ultralong protocol | 37 (7.33%) | 59 (6.28%) | 92 (3.75%) |
| Long protocol | 65 (12.87%) | 82 (8.73%) | 100 (4.08%) |
| Short protocol | 89 (17.62%) | 118 (12.57%) | 191 (7.79%) |
| GnRH agonist | 43 (8.51%) | 95 (10.12%) | 323 (13.18%) |
| PPOS related | 26 (5.15%) | 40 (4.26%) | 65 (2.65%) |
| Others | 16 (3.17%) | 131 (13.95%) | 618 (25.21%) |
| IVF/ICSI, n (%) ^*^ |  |  |  |
| IVF | 235 (46.53%) | 327 (34.82%) | 671 (27.38%) |
| ICSI | 207 (40.99%) | 418 (44.52%) | 848 (34.6%) |
| others | 63 (12.48%) | 194 (20.66%) | 932 (38.03%) |
| Number of initial oocyte retrieval per women (n) | 4.00 (2.00, 5.00) ^a^ | 2.00 (1.00, 4.00) ^b^ | 2.00 (1.00, 3.00) ^c^ |
| Total oocytes retrieved per women (n) | 6.00 (4.00, 8.00) ^a^ | 12.00 (7.00, 16.00) ^b^ | 13.00 (9.00, 18.00) ^c^ |
| Total embryos retrieved per women (n) | 3.00 (2.00, 4.00) ^a^ | 5.00 (3.00, 6.00) ^b^ | 6.00 (4.00, 7.00) ^c^ |
| Cleavage embryos (n) | 1.00 (0.00, 2.00) ^a^ | 2.00 (1.00, 4.00) ^b^ | 4.00 (2.00, 6.00) ^c^ |
| Blast embryos (n) | 2.00 (0.00, 3.00) ^a^ | 2.00 (0.00, 4.00) ^a^ | 1.00 (0.00, 3.00) ^b^ |
| FET cycles per women (n) | 1.00 (1.00, 2.00) ^a^ | 2.00 (1.00, 3.00) ^b^ | 2.00 (1.00, 3.00) ^c^ |
| FET protocol proportion, n (%) | |  |  |
| Artificial cycle | 163 (44.54%) | 204 (43.87%) | 305 (42.78%) |
| Natural cycle | 65 (17.76%) | 83 (17.85%) | 139 (19.5%) |
| Ovulation induction cycle | 79 (21.58%) | 84 (18.06%) | 150 (21.04%) |
| Down-regulation cycle | 59 (16.12%) | 94 (20.22%) | 119 (16.69%) |
| Average number of OPU cycles per embryo transfer | 1.38 | 2.01 | 3.43 |
| First OPU- last transfer interval | 3.50 (2.00, 6.00) ^a^ | 12 (9.00, 16.00) ^b^ | 19.00 (12.00, 28.00) ^c^ |
| First OPU-first clinical pregnancy interval | 3.00 (2.00, 6.00) ^a^ | 9.00 (5.00, 13.00) ^b^ | 14.00 (9.00, 23.00) ^c^ |
| Number of embryo transfers to first clinical pregnancy | 1.00 (1.00, 1.00) ^a^ | 1.00 (1.00, 2.00) ^b^ | 2.00 (1.00, 2.00) ^c^ |
| Number of embryos to first clinical pregnancy | 0.00 (0.00, 2.00) | 0.00 (0.00, 2.00) | 0.00 (0.00, 2.00) |
| First OPU- first live birth interval | 3.50 (2.00, 6.00) ^a^ | 11.00 (7.00, 13.00) ^b^ | 14.00 (10.00, 24.00) ^c^ |
| Number of embryo transfers to live birth | 1.00 (1.00, 2.00) ^a^ | 2.00 (1.00, 3.00) ^b^ | 2.00 (1.00, 3.00) ^b^ |
| Number of embryos to live birth | 0.00 (0.00, 2.00) ^a^ | 0.00 (0.00, 2.00) ^a^ | 0.00 (0.00, 0.00) ^b^ |

The characteristics of women in different OPU cycle groups. Non-normally distributed continuous data are presented as median (interquartile range, IQR) and compared using the Kruskal-Wallis test. Categorical variables are expressed as number (%) and compared using the chi-square or Fisher’s exact test. For post-hoc pairwise comparisons, the Bonferroni correction was applied. Different superscript letters (a, b and c) and asterisk (*) indicate statistically significant differences between groups at *P* < 0.05. BMI, body mass index; OPU, oocyte pick-up; GnRH, gonadotropin-releasing hormone; PPOS, progestin-primed ovarian stimulation; IVF, *in vitro* fertilization; ICSI, intracytoplasmic sperm injection; ‌FET, frozen embryo transfer.

**Appendix Table5**

| Total oocyte retrieval groups | Group1 <6 (n=294) | Group2 6-10 (n=284) | Group3 ≥11 (n=321) |
| --- | --- | --- | --- |
| Age (years) | 36.00 (32.00, 40.00) ^a^ | 35.00 (31.00, 39.00) ^a^ | 34.00 (31.00, 38.00) ^b^ |
| BMI (kg/m^2^) | 23.44 (21.23, 25.71) | 23.44 (21.48, 26.17) | 23.05 (20.83, 25.40) |
| Number of women having different OPU cycles ^*^ |  |  |  |
| 1 | 108 (36.73%) | 33 (11.62%) | 0 (0.00%) |
| 2 | 67 (22.79%) | 73 (25.70%) | 42 (13.08%) |
| 3 | 42 (14.29%) | 40 (14.08%) | 59 (18.38%) |
| 4 | 33 (11.22%) | 40 (14.08%) | 56 (17.45%) |
| 5 | 24 (8.16%) | 30 (10.56%) | 35 (10.90%) |
| ≥6 | 20 (6.80%) | 68 (23.94%) | 129 (40.19%) |
| Time duration of total OPU (months） | 2.00 (0.00, 7.00) ^a^ | 6.50 (3.00, 14.00) ^b^ | 16.00 (8.00, 27.00) ^c^ |
| Number of women on different ovulation induction protocols, n (%) ^*^ | |  |  |
| GnRH antagonist | 289 (37.83%) | 385 (31.35%) | 577 (30.32%) |
| Mild stimulation | 109 (14.27%) | 139 (11.32%) | 206 (10.83%) |
| Ultralong protocol | 18 (2.36%) | 51 (4.15%) | 119 (6.25%) |
| Long protocol | 33 (4.32%) | 71 (5.78%) | 143 (7.51%) |
| Short protocol | 80 (10.47%) | 109 (8.88%) | 209 (10.98%) |
| GnRH agonist | 57 (7.46%) | 140 (11.40%) | 264 (13.87%) |
| PPOS related | 29 (3.80%) | 40 (3.26%) | 62 (3.26%) |
| Others | 149 (19.50%) | 239 (23.86%) | 323 (16.97%) |
| IVF/ICSI, n (%) ^*^ |  |  |  |
| IVF | 255 (33.38%) | 383 (31.19%) | 595 (31.27%) |
| ICSI | 178 (23.30%) | 394 (32.08%) | 901 (47.35%) |
| others | 331 (43.32%) | 451 (36.73%) | 407 (21.39%) |
| Number of initial oocyte retrieval per women (n) | 2.00 (1.00, 3.00) ^a^ | 3.00 (1.00, 4.00) ^b^ | 4.00 (2.00, 6.00) ^c^ |
| Total embryos retrieved per women (n) | 2.00 (1.00, 2.00) ^a^ | 2.00 (1.00, 3.00) ^b^ | 3.00 (2.00, 5.00) ^c^ |
| Cleavage embryos (n) | 1.00 (0.00, 2.00) ^a^ | 0.00 (0.00, 2.00) ^b^ | 2.00 (0.00, 4.00) ^c^ |
| Blast embryos (n) | 0.00 (0.00, 1.00) ^a^ | 1.00 (0.00, 2.00) ^b^ | 1.00 (0.00, 2.00) ^c^ |
| FET cycles per women (n) | 1.00 (1.00, 1.00) ^a^ | 1.00 (1.00, 2.00) ^b^ | 1.00 (1.00, 2.00) ^c^ |
| FET protocol proportion, n (%) ^*^ |  |  |  |
| Artificial cycle | 91 (39.22%) | 194 (46.19%) | 387 (43.39%) |
| Natural cycle | 56 (24.14%) | 72 (17.14%) | 159 (17.83%) |
| Ovulation induction cycle | 56 (24.14%) | 75 (17.86%) | 182 (20.40%) |
| Down-regulation cycle | 29 (12.50%) | 79 (18.81%) | 164 (18.39%) |
| Average number of OPU cycles per embryo transfer | 3.28 | 2.92 | 2.13 |
| First OPU- last transfer interval | 4.00 (2.00, 8.00) ^a^ | 9.00 (5.00, 14.00) ^b^ | 16.00 (10.00, 26.00) ^c^ |
| First OPU-first clinical pregnancy interval | 3.00 (2.00, 5.50) ^a^ | 6.00 (3.00, 10.00) ^b^ | 12.00 (5.00, 19.00) ^c^ |
| Number of embryo transfers to first clinical pregnancy | 1.00 (1.00, 1.00) ^a^ | 1.00 (1.00, 2.00) ^b^ | 2.00 (1.00, 3.00) ^c^ |
| Number of embryos to first clinical pregnancy | 0.00 (0.00, 1.00) ^a^ | 0.00 (0.00, 2.00) ^b^ | 0.00 (0.00, 3.00) ^c^ |
| First OPU- first live birth interval | 3.50 (2.00, 6.00) ^a^ | 7.00 (3.00, 11.00) ^b^ | 13.00 (8.00, 19.00) ^c^ |
| Number of embryo transfers to live birth | 1.00 (1.00, 1.00) ^a^ | 1.00 (1.00, 2.00) ^b^ | 2.00 (1.00, 3.00) ^c^ |
| Number of embryos to live birth | 0.00 (0.00, 0.00) ^a^ | 0.00 (0.00, 2.00) ^b^ | 0.00 (0.00, 2.00) ^b^ |

The characteristics of women in different total oocyte retrieval groups. Non-normally distributed continuous data are presented as median (interquartile range, IQR) and compared using the Kruskal-Wallis test. Categorical variables are expressed as number (%) and compared using the chi-square or Fisher’s exact test. For post-hoc pairwise comparisons, the Bonferroni correction was applied. Different superscript letters (a, b and c) and asterisk (*) indicate statistically significant differences between groups at *P* < 0.05. BMI, body mass index; OPU, oocyte pick-up; GnRH, gonadotropin-releasing hormone; PPOS, progestin-primed ovarian stimulation; IVF, *in vitro* fertilization; ICSI, intracytoplasmic sperm injection; ‌FET, frozen embryo transfer.
